# Angioplastia do Tronco da Artéria Coronária Esquerda no Tratamento de Compressão Extrínseca em Pacientes com Hipertensão Pulmonar

**DOI:** 10.36660/abc.20250002

**Published:** 2025-07-10

**Authors:** Luciana Dornfeld Bichuette, Daniela Calderaro, Pedro Alves Lemos, Eduardo Kaiser Ururahy Nunes Fonseca, Luiza Sarmento Tatagiba, Tulio Martins Vieira, Yally Priscila Pessoa Nascimento, Marcela Araujo Castro, Taysa Antônia Félix Silva, Yuri de Deus Montalverne Parente, Caio Julio Fernandes, Francisco Akira Malta Cardozo, Cesar Higa Nomura, Pedro Henrique Almeida Marins, Rogerio Souza, Carlos Vianna Poyares Jardim

**Affiliations:** 1 Instituto do Coração do Hospital das Clínicas Faculdade de Medicina Universidade de São Paulo São Paulo SP Brasil Instituto do Coração do Hospital das Clínicas da Faculdade de Medicina da Universidade de São Paulo, São Paulo, SP – Brasil

**Keywords:** Hipertensão Pulmonar, Angioplastia, Vasos Coronários

## Abstract

**Fundamento:**

A apresentação clínica mais comum da hipertensão pulmonar (HP) inclui dispneia aos esforços, sintomas de congestão sistêmica e síncope. A angina pectoris também pode ser uma manifestação relevante, especialmente em casos nos quais ocorre compressão extrínseca do tronco da artéria coronária esquerda (TCE) pela artéria pulmonar dilatada. Contudo, persistem lacunas significativas quanto às estratégias diagnósticas e terapêuticas mais adequadas para a obstrução coronariana nesse contexto clínico.

**Objetivos:**

Avaliar a viabilidade e o impacto da angioplastia coronariana com stent no alívio dos sintomas em pacientes com HP associada à compressão extrínseca do TCE.

**Métodos:**

Trata-se de um estudo descritivo que incluiu 12 pacientes com HP acompanhados no Ambulatório de Circulação Pulmonar do Instituto do Coração do Hospital das Clínicas da Faculdade de Medicina da Universidade de São Paulo. Todos foram submetidos a angioplastia coronariana com implante de stent visando ao tratamento da compressão extrínseca do TCE.

**Resultados:**

Foram analisados 12 pacientes, com idade média de 47,9 anos, predominantemente com HP do grupo 1 e em uso de terapia específica. Todos apresentaram excelente resultado imediato da angioplastia, com alívio da angina após 30 dias. Durante o seguimento médio de 33 meses, não foram observadas complicações relacionadas ao procedimento, mantendo-se o controle dos sintomas anginosos. Quatro pacientes evoluíram a óbito em decorrência da progressão da insuficiência cardíaca.

**Conclusão:**

Os resultados observados reforçam a viabilidade da angioplastia coronariana como estratégia para o alívio sintomático da angina em pacientes com compressão do TCE e HP. Estudos adicionais são necessários para avaliar o impacto dessa intervenção em desfechos clínicos robustos, bem como a relevância do rastreamento em pacientes assintomáticos.

## Graphical abstract

**Figura Central:**
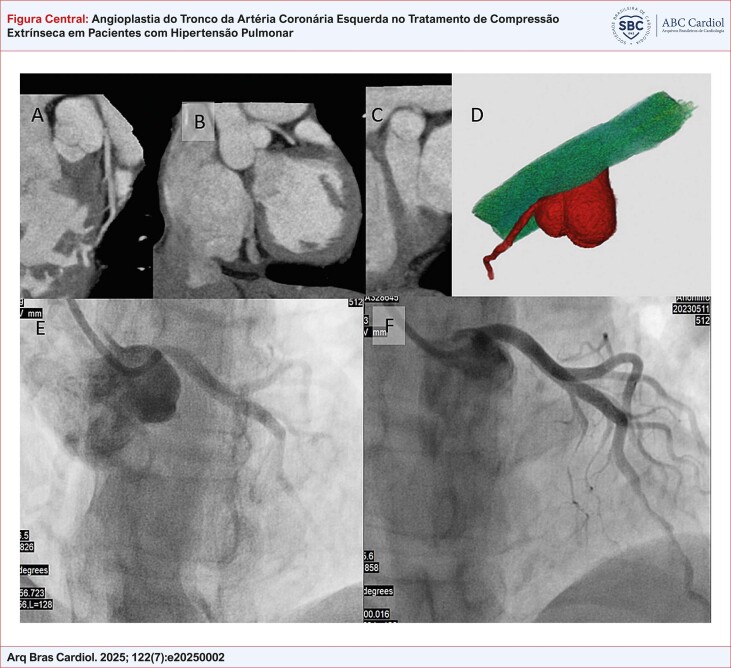
: Angioplastia do Tronco da Artéria Coronária Esquerda no Tratamento de Compressão Extrínseca em Pacientes com Hipertensão Pulmonar

Ilustração de caso clínico (paciente número 12). Imagens da angiotomografia evidenciando, em (A), reconstrução curva; em (B), eixo longo sagital do vaso; e em (C), eixo curto do vaso, demonstrando sinais de compressão com redução luminal significativa do tronco da artéria coronária esquerda (TCE). Em (D), reformatação tridimensional ilustrando a compressão do TCE pelo tronco da artéria pulmonar. Em (E), angiografia do TCE antes do procedimento. Em (F), angiografia do mesmo segmento após angioplastia com implante de stent.

## Introdução

A hipertensão pulmonar (HP) é uma condição de elevada gravidade e impacto prognóstico, caracterizada por pressão arterial pulmonar média superior a 20 mmHg em repouso, mensurada por meio de cateterismo cardíaco direito. Essa condição pode ser classificada em cinco grupos, conforme o mecanismo fisiopatológico predominante.^1,2^ Em escala global, as cardiopatias esquerdas constituem a principal causa de HP, seguidas pelas doenças pulmonares.^3^ Contudo, independentemente do mecanismo fisiopatológico envolvido, a elevação da pressão no leito vascular pulmonar associa-se invariavelmente a piora do prognóstico.^4,5^

A mortalidade em pacientes com HP é, predominantemente, consequência da falência do ventrículo direito, levando à insuficiência cardíaca (IC) progressiva. No entanto, até 25% dos óbitos ocorrem em decorrência de morte súbita.^6,7^ Em relação às manifestações clínicas, a dispneia aos esforços configura-se como o sintoma mais prevalente, embora sinais de congestão sistêmica e episódios de síncope também sejam frequentemente observados.

No contexto da HP, a angina é relatada por aproximadamente 16% a 29% dos pacientes,^8,9^ podendo resultar do desequilíbrio entre a oferta e o consumo de oxigênio pelo miocárdio, mesmo na ausência de alterações no fluxo das artérias coronárias epicárdicas. O aumento da tensão da parede do ventrículo direito leva à redução da reserva de fluxo coronariano, agravada pelo aumento do consumo de oxigênio decorrente da sobrecarga hemodinâmica e hipertrofia ventricular, mecanismos suficientes para justificar a ocorrência de isquemia.^10^ Adicionalmente, a dilatação da artéria pulmonar, observada em até 76,6% dos pacientes com HP grave,^11,12^ pode provocar compressão do tronco da artéria coronária esquerda (TCE), contribuindo para a manifestação de angina.^13^ O aumento do diâmetro da artéria pulmonar associa-se a maior risco de morte súbita em pacientes com HP, sendo a obstrução do TCE considerada um dos possíveis mecanismos envolvidos nesse desfecho.^14^

A abordagem ideal diante do diagnóstico de compressão extrínseca do TCE permanece indefinida. No entanto, séries de casos recentes têm demonstrado resultados promissores com a realização de angioplastia coronariana percutânea.^15,16^ Com o intuito de aprimorar a assistência em nosso centro de referência para HP, implementamos um fluxograma assistencial para investigação de angina e seleção de casos candidatos à angioplastia coronária, mediante discussão multidisciplinar. O presente estudo descreve nossa experiência inicial com 12 pacientes submetidos a esse protocolo terapêutico.

## Métodos

### Seleção dos pacientes

Trata-se de uma série de casos retrospectiva, incluindo 12 pacientes consecutivos com HP atendidos no Ambulatório de Circulação Pulmonar do Instituto do Coração (InCor) do Hospital das Clínicas da Faculdade de Medicina da Universidade de São Paulo (HCFMUSP), submetidos a angioplastia coronária com implante de *stent* para tratamento de compressão extrínseca do TCE. Atualmente, aproximadamente mil pacientes com HP realizam seguimento ambulatorial no InCor, sendo avaliados de forma integrada por pneumologistas, cardiologistas e reumatologistas. Mediante consenso multidisciplinar, pacientes com história de angina e/ou evidências de acometimento de câmaras cardíacas esquerdas são encaminhados para investigação de compressão extrínseca do TCE.

A investigação diagnóstica é realizada por meio de estudo anatômico, preferencialmente utilizando angiotomografia de artérias coronárias (ATC). Este relato baseia-se em registro retrospectivo da prática assistencial do serviço, descrevendo os primeiros casos tratados. Por isso, não foi aplicado Termo de Consentimento Livre e Esclarecido. Em todas as etapas do estudo, a preservação da confidencialidade e anonimização dos dados dos pacientes foram garantidas. O protocolo de avaliação dos pacientes com HP encaminhados para cateterismo cardíaco foi aprovado pela Comissão de Ética em Pesquisa do HCFMUSP sob o CAAE nº 11032919.8.0000.0068.

### ATC

As ATCs foram realizadas em tomógrafo de 320 detectores (Aquilion ONE, Canon Medical Systems, Japão), seguindo protocolo de aquisição padrão, sem o uso de medicações para controle da frequência cardíaca ou vasodilatação. Embora tais medicações sejam rotineiramente empregadas em protocolos de avaliação de doença coronariana obstrutiva, na população do presente estudo, composta majoritariamente por pacientes em uso de sildenafila, os nitratos são formalmente contraindicados e os betabloqueadores apresentam baixa tolerabilidade.

**Figura 1:**
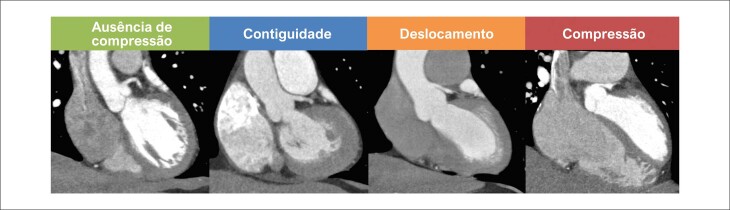
– Classificação tomográfica adaptada de Galie et al. Representação da relação anatômica entre o tronco da artéria coronária esquerda e o tronco da artéria pulmonar: ausência de compressão; contiguidade; deslocamento; compressão com estenose significativa.

O processamento e a análise das imagens de ATC foram realizados por um único especialista em imagem cardiovascular não invasiva. A presença de compressão extrínseca do TCE pelo tronco da artéria pulmonar foi avaliada e classificada segundo os critérios propostos por Galie et al. (Esta citação não consta na lista de referências. Favor informar) conforme descrito a seguir: (1) ausência de compressão ou deslocamento do TCE; (2) contiguidade, definida como distância igual ou inferior a 1 mm entre o tronco da artéria pulmonar e o TCE, sem evidência de deslocamento ou estenose significativa; (3) deslocamento, caracterizado por desvio do trajeto do TCE provocado pela artéria pulmonar, com ângulo de origem inferior a 60º, mas sem redução luminal significativa (≥50%); e (4) estenose significativa, definida pela presença de compressão com redução luminal ≥50% do TCE. A classificação adaptada utilizada neste estudo está ilustrada na Figura 1. Adicionalmente, foi mensurado o diâmetro transverso do tronco da artéria pulmonar.

### Angiografia e intervenção coronariana percutânea (ICP)

A angiografia coronária foi realizada por acesso radial ou femoral, conforme critério do intervencionista responsável. As projeções angiográficas foram selecionadas de acordo com as recomendações previamente descritas por estudos da literatura,^17^ incluindo injeções com ou sem cateterização seletiva do TCE.

A ICP pôde ser realizada de forma ad hoc ou em tempo estadiado. O procedimento foi conduzido sob anticoagulação plena com heparina não fracionada, titulada para manter o tempo de coagulação ativado superior a 250 segundos. A escolha do tipo e das dimensões do *stent* intracoronário, bem como da estratégia intervencionista, ficou a critério do operador, considerando a angiografia basal, com apoio de quantificação angiográfica online, e a eventual utilização de imagem intravascular, quando disponível, não sendo esta obrigatória.

De maneira geral, a técnica adotada baseou-se no implante direto do *stent* intracoronário, sem necessidade de pré-dilatação, com posicionamento desde o óstio do TCE, evitando-se a ultrapassagem da bifurcação. A realização de pós-dilatação foi considerada opcional, a depender da avaliação do operador.

Após o procedimento, todos os pacientes permaneceram em uso de terapia antiplaquetária conforme protocolo assistencial vigente.

### Seguimento dos pacientes

Os pacientes foram avaliados 1 mês após a intervenção e, subsequentemente, em intervalos de 4 a 6 meses. Durante o seguimento, foram monitorados a evolução clínica da HP, a classe funcional de IC, a persistência ou recorrência de angina e a ocorrência de óbitos. Adicionalmente, foi registrada a duração da terapia antitrombótica prescrita, incluindo dupla antiagregação plaquetária ou combinação de anticoagulação oral com clopidogrel.

### Análise estatística

Trata-se de um estudo descritivo, no qual as variáveis contínuas foram expressas como média ± desvio-padrão, enquanto as variáveis categóricas foram apresentadas em frequências absolutas e relativas. Em virtude do delineamento observacional e do tamanho amostral reduzido (n=12), não foram realizadas análises inferenciais, como testes de hipóteses ou comparações entre grupos. As análises estatísticas foram conduzidas por meio do software Microsoft Excel; os resultados foram interpretados a partir de descrições numéricas simples.

## Resultados

Foram incluídos 12 pacientes, sendo sete do sexo feminino e cinco do sexo masculino, com idade média de 47,9 ± 15,0 anos. Desses, 11 apresentavam diagnóstico de hipertensão arterial pulmonar (HAP) e um paciente apresentava HP secundária a IC com fração de ejeção do ventrículo esquerdo preservada.

Antes da realização da angioplastia coronariana, a estratificação de risco, conforme a estratégia derivada do *Comparative, Prospective Registry of Newly Initiated Therapies for Pulmonary Hypertension* (COMPERA),^18^ identificou dois pacientes em alto risco, cinco em risco intermediário e cinco em baixo risco.

A maioria dos pacientes encontrava-se em uso de terapia específica combinando sildenafila e ambrisentana, conforme a disponibilidade de acesso à medicação no período das indicações terapêuticas. As características clínicas detalhadas da amostra estão apresentadas na Tabela 1.

**Tabela 1:** – Características clínicas e hemodinâmicas pré-angioplastia coronária dos pacientes

ID do estudo	Sexo	Idade (anos)	Etiologia	Estratificação de risco	PMAP (mmHg)	RVP (UW)	DC (l/min)	AP (mm)	BNP (pg/ml)	CF	Motivo da investigação coronariana
1	F	47	Esquistossomose	Baixo	70	9,6	5,6	44	209	2	Angina
2	M	66	HAP	Intermediário	58	11,2	3,9	52	187	3	Disfunção de VE
3	M	55	HAP	Intermediário	50	7,6	4,6	81	62	3	Angina
4	F	35	CC	Baixo	73	14,8	4,6	48	38	2	Angina
5	M	78	HAP	Intermediário	62	10,8	4,8	62	104	3	Angina
6	F	40	CC	Intermediário	55	2,9	15,3	41	152	2	Angina
7	F	29	HAP	Alto	66	14,5	3,5	49	185	4	Angina
8	F	37	CC	Baixo	70	9,8	6,3	54	291	2	Angina
9	F	52	Esquistossomose	Alto	72	18,6	3,0	108	1026	4	Choque cardiogênico
10	M	63	HP grupo 2	Baixo	58	8,0	4,7	59	98	2	ICFEP
11	F	35	HAP	Intermediário	67	13,6	3,9	41	56	3	Angina
12	M	38	CC	Baixo	61	11,9	4,6	34	36	3	Angina

AP: artéria pulmonar; diâmetro medido em milímetros (mm); BNP: peptídeo natriurético atrial, em pg/ml; CC: cardiopatia congênita; CF: classe funcional da insuficiência cardíaca, conforme classificação da New York Heart Association; DC: débito cardíaco, em litros por minuto (l/min); HAP: hipertensão arterial pulmonar; ICFEP: insuficiência cardíaca com fração de ejeção preservada; PCP: pressão capilar pulmonar, em milímetros de mercúrio (mmHg); PMAP: pressão média da artéria pulmonar, em milímetros de mercúrio (mmHg); RVP: resistência vascular pulmonar, em unidades Wood (UW); VE: ventrículo esquerdo.

A principal indicação para avaliação coronariana foi a presença de angina. A ATC foi utilizada como exame de triagem preferencial em 10 dos 12 casos, enquanto a coronariografia confirmou, em todos os pacientes, a presença de compressão com estenose significativa do TCE. Apenas um paciente apresentava aterosclerose coronariana concomitante, localizada em outro segmento da árvore coronariana, sem obstrução significativa.

**Figura 2:**
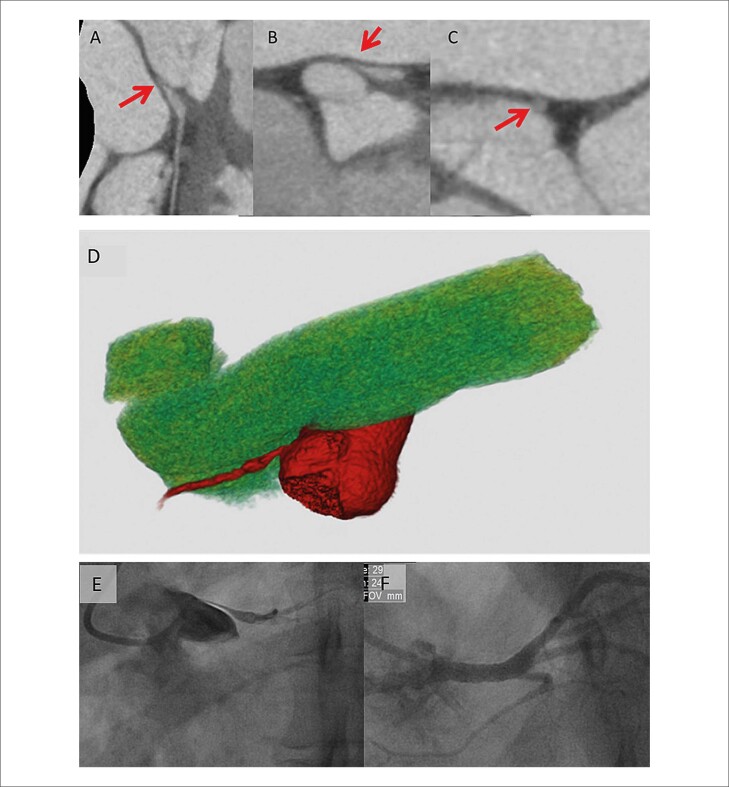
– Ilustração do caso clínico (paciente número 8). Imagens da angiotomografia de coronárias demonstrando, em (A), reconstrução curva; em (B), eixo longo sagital do vaso; e em (C), eixo curto do vaso, evidenciando sinais de compressão com redução luminal significativa do tronco da artéria coronária esquerda (TCE) (setas vermelhas). Em (D), reformatação tridimensional mostrando a compressão do TCE pela artéria pulmonar. Em (E), angiografia do TCE antes do procedimento. Em (F), angiografia após angioplastia com implante de stent.

Foram implantados *stents* farmacológicos em oito pacientes e *stents* convencionais em quatro, com diâmetros variando entre 3,5 mm, 4,0 mm e 5,0 mm, e comprimentos entre 12 mm e 20 mm, ajustados à extensão da obstrução e à disponibilidade de materiais no serviço. Obteve-se, em todos os casos, bom resultado primário (Figura 2 e Figura Central).

Em nove pacientes, a implantação de um único *stent* foi suficiente para a resolução da obstrução; entretanto, em três casos, o resultado após o primeiro *stent* foi considerado subótimo, sendo necessária a colocação de uma segunda malha para reforço da força radial e sustentação contra a compressão extrínseca. A realização de pós-dilatação foi opcional, porém realizada na maioria dos casos, especialmente quando não foi possível restringir o *stent* exclusivamente ao TCE.

**Figura 3:**
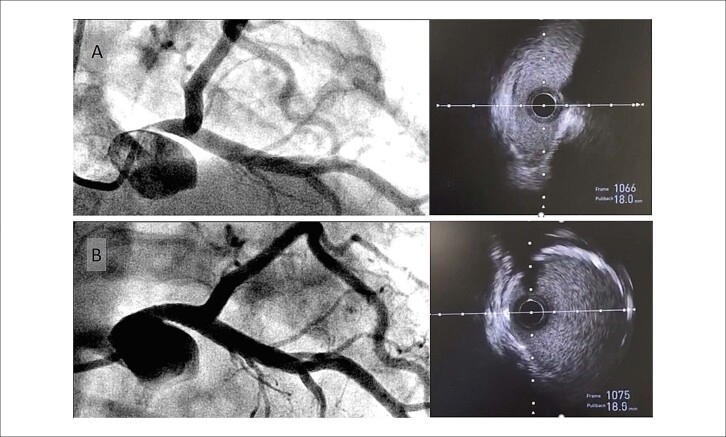
– Cineangiocoronariografia e ultrassom intracoronário antes e após o implante do stent no tronco da artéria coronária esquerda (paciente número 5). Na avaliação basal (A), observa-se redução luminal acentuada tanto na cineangiografia (esquerda) quanto no ultrassom intracoronário (direita), evidenciando luz em fenda, sem presença de placa aterosclerótica. Após o implante do stent (B), verifica-se restauração das dimensões e da simetria da luz vascular.

Nos casos em que a liberação do *stent* avançou do TCE em direção à artéria descendente anterior, foi adotada a técnica provisional, com otimização exclusiva da dilatação do segmento proximal do *stent*, preservando a bifurcação. Detalhes adicionais relacionados às intervenções estão apresentados na Tabela 2. O ultrassom intracoronário foi utilizado em dois casos para guiar a angioplastia (Figura 3).

**Tabela 2:** – Detalhes das intervenções de angioplastia coronária dos pacientes

ID do estudo	Número de stents	Tipo de stent	Espessura da haste do stent	Tamanho do stent	Técnica empregada	Pós-dilatação	Uso de IVUS
1	1	Convencional	—	5,0 × 15 mm	TCE isolado	Não	Não
2	1	Farmacológico	81 μm	4,0 × 12 mm	TCE isolado	Sim	Sim
3	1	Convencional	81 μm	4,0 × 16 mm	TCE isolado	Não	Não
4	1	Convencional	97 μm	3,5 × 20 mm	TCE-DA	Sim	Não
5	1	Farmacológico	81 μm	4,0 × 20 mm	TCE-DA	Sim	Sim
6	2	Convencional + Convencional	80 μm	4,0 × 8 e 4,0 × 9 mm	TCE-DA	Sim	Não
7	1	Farmacológico	75 μm	3,5 × 23mm	TCE-DA	Sim	Não
8	1	Farmacológico	65 μm	3,5 × 16 mm	TCE-DA	Sim	Não
9	2	Farmacológico + Convencional	86 μm	4,0 × 15 e 4,0 × 12 mm	TCE isolado	Sim	Não
10	1	Farmacológico	81 μm	4,0 × 12 mm	TCE isolado	Sim	Não
11	2	Farmacológico + Farmacológico	75 μm	4,0 × 13 e 4,0 × 15 mm	TCE isolado	Sim	Não
12	1	Farmacológico	75 μm	4,0 × 16 mm	TCE-DA	Sim	Não

DA: artéria descendente anterior; IVUS: ultrassom intracoronário; TCE: tronco da artéria coronária esquerda.

**Figura 4:**
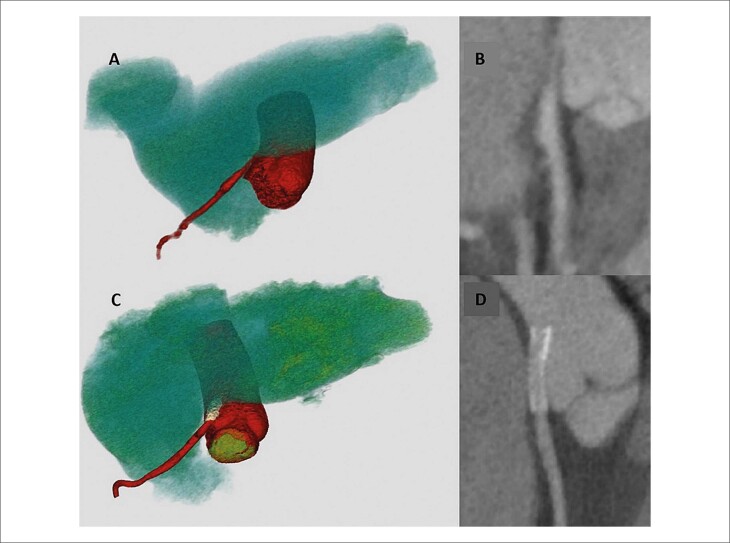
– Angiotomografia de coronárias (paciente número 4) antes e após angioplastia do tronco da artéria coronária esquerda (TCE) com implante de stent. Na linha superior (A e B), imagens pré-angioplastia demonstram redução luminal importante na origem do TCE, decorrente de compressão extrínseca pelo tronco da artéria pulmonar. Na linha inferior (C e D), observam-se as imagens pós-angioplastia, evidenciando o stent recobrindo o TCE desde o óstio, com luz preservada e sem sinais de estenose residual. As imagens foram obtidas por reconstrução tridimensional (A e C) e reformatação curva (B e D).

Todos os pacientes apresentaram melhora do quadro de angina após a realização da angioplastia coronária, com resolução completa dos sintomas em oito casos e alívio significativo em um paciente. No período subsequente ao primeiro ano, quatro pacientes foram submetidos à reavaliação não invasiva da patência do *stent*, por meio de ATC, a qual demonstrou manutenção do resultado satisfatório em todos os casos (Figura 4). Essa prática foi adotada de forma individualizada, conforme decisão do médico assistente.

A terapia antiplaquetária dupla foi mantida por 10±7 meses, sem registro de eventos hemorrágicos. No seguimento médio de 33 meses, observou-se necessidade de escalonamento da terapia específica para HP em 50% dos pacientes, em virtude da progressão da doença. Durante o acompanhamento, dois pacientes faleceram por descompensação da HP (após 12 e 18 meses da angioplastia) e dois faleceram em domicílio por morte súbita (após 7 e 47 meses da angioplastia), todos com progressão da HP e já classificados em risco intermediário ou alto no momento da ICP.

## Discussão

Este estudo representa a primeira série de casos publicada no Brasil abordando o tratamento da compressão extrínseca do TCE em pacientes com HP. O prognóstico dessa condição permanece pouco elucidado e, possivelmente, não é diretamente comparável ao da obstrução aterosclerótica do TCE. Embora seja uma causa reconhecida de angina, seu impacto sobre a mortalidade ainda não foi claramente estabelecido. Considerando que aproximadamente 25% dos óbitos em pacientes com HP decorrem de morte súbita,^6,14^ é plausível que parte desses eventos possa estar relacionada à compressão extrínseca do TCE — uma complicação potencialmente tratável e provavelmente subdiagnosticada.

O intervalo entre o início dos sintomas e a confirmação do diagnóstico de HP costuma ser superior a 2 anos, período em que a maioria dos pacientes já apresenta doença em estágio avançado.^19^ Como consequência, observa-se frequentemente dilatação significativa das artérias pulmonares, que, devido à sua proximidade anatômica, podem deslocar o TCE em direção ao seio coronário, resultando em redução do calibre do seu óstio. Estudos relatam prevalência de até 40% de obstrução significativa do TCE por compressão extrínseca em pacientes com HP e angina.^8^ Em uma série anterior do InCor, Mesquita et al. avaliaram 36 pacientes com HP do grupo 1 e cardiopatia congênita submetidos a cineangiocoronariografia, identificando compressão superior a 50% do TCE em sete casos. Os principais preditores de compressão identificados foram o diâmetro da artéria pulmonar superior a 40 mm e a maior razão artéria pulmonar/aorta.^20^ Achados semelhantes foram descritos por Galiè et al., que relataram sensibilidade de 83% e especificidade de 70% para o critério de diâmetro da artéria pulmonar superior a 40 mm.^8^ Adicionalmente, Akbal et al. demonstraram que a gravidade da HP, avaliada por maiores valores de pressão média da artéria pulmonar e resistência vascular pulmonar, também se associa a maior risco de compressão do TCE.^15^ Na presente série, apenas um paciente apresentava diâmetro da artéria pulmonar inferior a 40 mm, sendo a média do diâmetro registrada de 56 mm (variando de 34 mm a 108 mm).

Em 2001, Kajita et al. descreveram o padrão angiográfico característico da compressão extrínseca do TCE, evidenciando aspecto de esmagamento do vaso entre a artéria pulmonar e a raiz da aorta. Nesse estudo, foi reportado um dos parâmetros diagnósticos mais utilizados atualmente: a acentuada angulação de saída do TCE, geralmente inferior a 39,5 graus. Além disso, foi estabelecido que a projeção oblíqua anterior esquerda cranial constitui a melhor incidência para avaliação angiográfica desses pacientes.^17^ Essa projeção foi, inclusive, a que proporcionou melhor definição diagnóstica em nossa série, diferindo das projeções habitualmente empregadas na avaliação de obstrução aterosclerótica do TCE.

Apesar dos relatos existentes, persistem diversas lacunas quanto à abordagem ideal desses pacientes, dado o número ainda limitado de casos publicados. Sabe-se que a cirurgia de revascularização miocárdica em pacientes com HP apresenta elevada mortalidade, sobretudo devido à disfunção do ventrículo direito no pós-operatório, cenário em que a ICP emergiu como alternativa terapêutica eficaz para o tratamento da compressão extrínseca do TCE.

Saia et al. publicaram a maior série de casos disponível, descrevendo 53 pacientes com HP e obstrução ≥50% do TCE por compressão extrínseca submetidos a angioplastia coronária.^16^ O resultado primário foi satisfatório em todos os procedimentos. As angiografias coronárias foram repetidas protocolarmente após 9 meses, sendo necessária reintervenção em cinco pacientes: quatro por hiperplasia neointimal e um por recoil do *stent*. Durante o seguimento médio de 4,5 anos, a mortalidade foi de 37,3% (n=17), sem registro de infarto agudo do miocárdio ou trombose do *stent*. Na presente série, a mortalidade observada foi de 33,3%, resultado comparável ao da série italiana, e condizente com a expectativa de vida relatada em pacientes com HAP.^7^

As limitações do presente estudo incluem seu delineamento observacional, sem a inclusão de grupo controle, o que limita a comparação direta dos resultados com outras modalidades terapêuticas ou estratégias de manejo. Ademais, o reduzido tamanho amostral, associado à natureza retrospectiva da análise e ao período relativamente curto de seguimento, não permite conclusões definitivas quanto à durabilidade dos resultados obtidos, tampouco sobre a incidência de complicações em longo prazo, como a reestenose do *stent*. Essas limitações devem ser cuidadosamente consideradas na interpretação dos achados. Estudos futuros, com maior número de pacientes e seguimento prolongado, serão fundamentais para esclarecer o impacto prognóstico dessa abordagem terapêutica.

## Conclusão

A compressão extrínseca do TCE pela dilatação da artéria pulmonar deve ser considerada como diagnóstico diferencial em pacientes com HP que apresentem angina ou disfunção ventricular esquerda. A ICP demonstrou ser uma estratégia viável em centros especializados no manejo de HP, com resultados angiográficos e clínicos favoráveis. No entanto, são necessários estudos adicionais para avaliar o impacto prognóstico dessa intervenção, assim como para esclarecer os potenciais benefícios do rastreamento sistemático de compressão coronariana em pacientes sem angina.
