# Supplementary material for: Angioplastia do Tronco da Artéria Coronária Esquerda no Tratamento de Compressão Extrínseca em Pacientes com Hipertensão Pulmonar
Source: Arq Bras Cardiol. 2025 Jul 10;122(7):e20250002. [Article in Portuguese] doi: 10.36660/abc.20250002 (PMC12266617; doi:10.36660/abc.20250002)
Supplement: Translation

# Translation: Left Main Coronary Artery Angioplasty for the Treatment of Extrinsic Compression in Patients with Pulmonary Hypertension

**DOI:** 10.36660/abc.20250002i

**Authors:** Luciana Dornfeld Bichuette, Daniela Calderaro, Pedro Alves Lemos, Eduardo Kaiser Ururahy Nunes Fonseca, Luiza Sarmento Tatagiba, Tulio Martins Vieira, Yally Priscila Pessoa Nascimento, Marcela Araujo Castro, Taysa Antônia Félix Silva, Yuri de Deus Montalverne Parente, Caio Julio Fernandes, Francisco Akira Malta Cardozo, Cesar Higa Nomura, Pedro Henrique Almeida Marins, Rogerio Souza, Carlos Vianna Poyares Jardim

**Affiliations:** 1 Brazil Instituto do Coração do Hospital das Clínicas da Faculdade de Medicina da Universidade de São Paulo, São Paulo, SP – Brazil

**Keywords:** Pulmonary Hypertension, Angioplasty, Coronary Vessels

## Abstract

**Background:**

The most common clinical presentation of pulmonary hypertension (PH) includes exertional dyspnea, signs of systemic congestion, and syncope. Angina pectoris can also be a relevant manifestation, especially in cases where the left main coronary artery (LMCA) is externally compressed by a dilated pulmonary artery. However, significant gaps remain regarding the most appropriate diagnostic and therapeutic strategies for coronary obstruction in this clinical scenario.

**Objectives:**

To assess the feasibility and impact of coronary angioplasty with stent implantation on symptom relief in patients with PH and extrinsic compression of LMCA.

**Methods:**

This descriptive study included 12 patients with PH who were followed at the Pulmonary Circulation Outpatient Clinic of the Instituto do Coração, Hospital das Clínicas da Faculdade de Medicina da Universidade de São Paulo. All patients underwent coronary angioplasty with stent implantation to treat extrinsic compression of LMCA.

**Results:**

A total of 12 patients were analyzed, with a mean age of 47.9 years, predominantly with group 1 PH and under specific therapy. All procedures achieved excellent immediate results, with angina relief observed at 30 days. During a mean follow-up of 33 months, no procedure-related complications were reported, and angina symptoms remained controlled. Four patients died due to progressive heart failure.

**Conclusion:**

The findings support the feasibility of coronary angioplasty as a strategy for symptomatic relief of angina in patients with LMCA compression associated with PH. Further studies are needed to evaluate the impact of this intervention on hard clinical outcomes, as well as the role of screening in asymptomatic patients.

## Introduction

Pulmonary hypertension (PH) is a serious condition with significant prognostic implications, defined by a mean pulmonary arterial pressure greater than 20 mmHg at rest, as measured by right heart catheterization. PH can be classified into five groups according to the predominant pathophysiological mechanism.^1,2^ Globally, left heart diseases are the leading cause of PH, followed by pulmonary diseases.^3^ Regardless of the underlying mechanism, elevated pressure in the pulmonary vascular bed is consistently associated with worse prognosis.^4,5^

Mortality in patients with PH is predominantly due to right ventricular failure, leading to progressive heart failure (HF). However, up to 25% of deaths occur as a result of sudden cardiac death.^6,7^ Regarding clinical manifestations, exertional dyspnea is the most common symptom, although signs of systemic congestion and episodes of syncope are also frequently observed.

In the context of PH, angina is reported in approximately 16% to 29% of patients,^8,9^ and may result from an imbalance between myocardial oxygen supply and demand, even in the absence of flow-limiting lesions in the epicardial coronary arteries. Increased right ventricular wall tension leads to a reduction in coronary flow reserve, which is further aggravated by elevated oxygen consumption due to hemodynamic overload and ventricular hypertrophy — mechanisms that are sufficient to explain the occurrence of ischemia.^10^ Additionally, pulmonary artery dilation, observed in up to 76.6% of patients with severe PH,^11,12^ may cause extrinsic compression of the left main coronary artery (LMCA), contributing to angina symptoms.^13^ Pulmonary artery enlargement is also associated with an increased risk of sudden cardiac death in patients with PH, with LMCA obstruction considered one of the potential mechanisms involved in this outcome.^14^

The optimal approach to managing extrinsic compression of the LMCA remains undefined. However, recent case series have shown promising results with percutaneous coronary angioplasty.^15,16^ In an effort to enhance care at our PH referral center, we implemented a care pathway for the evaluation of angina and the selection of candidates for coronary angioplasty through a multidisciplinary discussion process. This study describes our initial experience with 12 patients who underwent this therapeutic protocol.

## Methods

### Patient selection

This is a retrospective case series including 12 consecutive patients with PH who were followed at the Pulmonary Circulation Outpatient Clinic of the Instituto do Coração (InCor), Hospital das Clínicas da Faculdade de Medicina da Universidade de São Paulo (HCFMUSP). All patients underwent coronary angioplasty with stent implantation to treat extrinsic compression of the LMCA. Currently, approximately 1,000 patients with PH are under outpatient follow-up at InCor, where they are assessed through an integrated approach by pulmonologists, cardiologists, and rheumatologists. Through a multidisciplinary consensus, patients presenting with a history of angina and/or evidence of left heart chamber involvement are referred for investigation of LMCA extrinsic compression.

Diagnostic assessment was performed through anatomical evaluation, preferably using coronary computed tomography angiography (CTA). This report is based on a retrospective review of the center’s clinical practice, describing the initial cases treated. Therefore, informed consent was not obtained. Patient confidentiality and data anonymization were maintained throughout all stages of the study. The protocol for evaluating PH patients referred for cardiac catheterization was approved by the Research Ethics Committee of HCFMUSP under CAAE no. 11032919.8.0000.0068.

### CTA

CTA examinations were performed using a 320-detector scanner (Aquilion ONE, Canon Medical Systems, Japan), following a standard acquisition protocol without the use of medications for heart rate control or vasodilation. Although such medications are routinely used in protocols for evaluating obstructive coronary artery disease, in the present study population — composed predominantly of patients receiving sildenafil — nitrates are formally contraindicated, and beta-blockers are poorly tolerated.

Image processing and analysis were conducted by a single specialist in non-invasive cardiovascular imaging. Extrinsic compression of the LMCA by the main pulmonary artery was assessed and classified according to the criteria proposed by Galie et al. as follows: (1) no compression or displacement of the LMCA; (2) contiguity, defined as a distance of 1 mm or less between the main pulmonary artery and the LMCA, without evidence of displacement or significant stenosis; (3) displacement, characterized by deviation of the LMCA course caused by the pulmonary artery, with an angle of origin less than 60°, but without significant luminal reduction (≥50%); and (4) significant stenosis, defined as compression with a luminal reduction of ≥50% of the LMCA. The adapted classification used in this study is illustrated in Figure 1. Additionally, the transverse diameter of the main pulmonary artery was measured.

**Figure 1:** – CT-based classification adapted from Galie et al. Illustration of the anatomical relationship between the left main coronary artery and the main pulmonary artery: no compression; contiguity; displacement; compression with significant stenosis.

### Coronary angiography and percutaneous coronary intervention (PCI)

Coronary angiography was performed via radial or femoral access, at the discretion of the attending interventional cardiologist. Angiographic projections were selected based on recommendations previously described in the literature,^17^ including injections with or without selective catheterization of LMCA.

PCI could be performed either ad hoc or in a staged approach. The procedure was conducted under full anticoagulation with unfractionated heparin, titrated to maintain an activated clotting time (ACT) above 250 seconds. The choice of intracoronary stent type and size, as well as the interventional strategy, was left to the operator’s discretion, based on baseline angiography, supported by online quantitative coronary analysis and the optional use of intravascular imaging when available, though not mandatory.

Overall, the technique involved direct stent implantation without prior balloon predilatation, positioning the stent from the LMCA ostium while avoiding extension beyond the bifurcation. Postdilation was considered optional, depending on the operator’s assessment.

Following the procedure, all patients remained on antiplatelet therapy according to the institution’s standard protocol.

### Patient follow-up

Patients were evaluated 1 month after the intervention and subsequently at intervals of 4 to 6 months. During follow-up, clinical progression of PH, HF functional class, persistence or recurrence of angina, and mortality were monitored. Additionally, the duration of prescribed antithrombotic therapy was recorded, including dual antiplatelet therapy or the combination of oral anticoagulation with clopidogrel.

### Statistical analysis

This was a descriptive study in which continuous variables were expressed as mean ± standard deviation, while categorical variables were presented as absolute and relative frequencies. Due to the observational design and small sample size (n=12), no inferential analyses, such as hypothesis testing or between-group comparisons, were performed. Statistical analyses were conducted using Microsoft Excel software, and results were interpreted through simple numerical descriptions.

## Results

A total of 12 patients were included, seven women and five men, with a mean age of 47.9 ± 15.0 years. Of these, 11 had a diagnosis of pulmonary arterial hypertension (PAH), while one patient had PH secondary to HF with preserved left ventricular ejection fraction.

Prior to coronary angioplasty, risk stratification based on the strategy derived from the Comparative, Prospective Registry of Newly Initiated Therapies for Pulmonary Hypertension (COMPERA)^18^ identified two patients at high risk, five at intermediate risk, and five at low risk.

Most patients were receiving combination therapy with sildenafil and ambrisentan, according to medication availability during the therapeutic indication period. Detailed clinical characteristics of the sample are presented in Table 1.

**Table 1:** – Clinical and hemodynamic characteristics of patients before coronary angioplasty

Study ID	Sex	Age (years)	Etiology	Risk stratification	mPAP (mmHg)	PVR (WU)	CO (L/min)	PA (mm)	BNP (pg/mL)	HF class	Reason for coronary evaluation
1	F	47	Schistosomiasis	Low	70	9.6	5.6	44	209	2	Angina
2	M	66	PAH	Intermediate	58	11.2	3.9	52	187	3	LV dysfunction
3	M	55	PAH	Intermediate	50	7.6	4.6	81	62	3	Angina
4	F	35	CHD	Low	73	14.8	4.6	48	38	2	Angina
5	M	78	PAH	Intermediate	62	10.8	4.8	62	104	3	Angina
6	F	40	CHD	Intermediate	55	2.9	15.3	41	152	2	Angina
7	F	29	PAH	High	66	14.5	3.5	49	185	4	Angina
8	F	37	CHD	Low	70	9.8	6.3	54	291	2	Angina
9	F	52	Schistosomiasis	High	72	18.6	3.0	108	1026	4	Cardiogenic shock
10	M	63	Group 2 PH	Low	58	8.0	4.7	59	98	2	HFpEF
11	F	35	PAH	Intermediate	67	13.6	3.9	41	56	3	Angina
12	M	38	CHD	Low	61	11.9	4.6	34	36	3	Angina

BNP: brain natriuretic peptide, in pg/mL; CHD: congenital heart disease; CO: cardiac output, in liters per minute (L/min); HF class: heart failure functional class, according to the New York Heart Association classification; HFpEF: heart failure with preserved ejection fraction; LV: left ventricle; mPAP: mean pulmonary artery pressure, in millimeters of mercury (mmHg); PA: pulmonary artery; diameter measured in millimeters (mm); PAH: pulmonary arterial hypertension; PVR: pulmonary vascular resistance, in Wood units (WU).

The main indication for coronary evaluation was the presence of angina. CTA was used as the preferred screening test in 10 of the 12 cases, while coronary angiography confirmed significant LMCA stenosis due to extrinsic compression in all patients. Only one patient had concomitant coronary atherosclerosis, located in another segment of the coronary tree, without significant obstruction.

Drug-eluting stents were implanted in eight patients and bare-metal stents in four, with diameters ranging from 3.5 mm, 4.0 mm, and 5.0 mm, and lengths between 12 mm and 20 mm, adjusted according to the extent of the obstruction and material availability at the institution. In all cases, a good primary result was achieved (Figure 2 and Central Illustration).

**Figure 2:** – Clinical case illustration (patient 8). Coronary computed tomography angiography images showing: (A) curved reconstruction; (B) sagittal long-axis view of the vessel; and (C) short-axis view of the vessel, highlighting signs of compression with significant luminal narrowing of left main coronary artery (LMCA) (red arrows). (D) Three-dimensional reconstruction showing LMCA compression by the pulmonary artery. (E) Angiography of LMCA before the procedure. (F) Angiography after stent implantation.

In nine patients, the implantation of a single stent was sufficient to resolve the obstruction; however, in three cases, the result after the first stent was considered suboptimal, requiring placement of a second stent to reinforce radial strength and support against extrinsic compression. Postdilation was optional but performed in most cases, particularly when it was not possible to limit the stent exclusively to the LMCA.

In cases where stent deployment extended from the LMCA toward the left anterior descending artery, the provisional technique was applied, with exclusive optimization of proximal stent dilation while preserving the bifurcation. Additional procedural details are presented in Table 2. Intravascular ultrasound was used in two cases to guide angioplasty (Figure 3).

**Table 2:** – Details of coronary angioplasty interventions in the study patients

Study ID	Number of stents	Type of stent	Stent strut thickness	Stent size	Technique used	Postdilation	Use of IVUS
1	1	Bare-metal	—	5.0 × 15 mm	Isolated LMCA	No	No
2	1	Drug-eluting	81 μm	4.0 × 12 mm	Isolated LMCA	Yes	Yes
3	1	Bare-metal	81 μm	4.0 × 16 mm	Isolated LMCA	No	No
4	1	Bare-metal	97 μm	3.5 × 20 mm	LMCA-LAD	Yes	No
5	1	Drug-eluting	81 μm	4.0 × 20 mm	LMCA-LAD	Yes	Yes
6	2	Bare-metal+ Bare-metal	80 μm	4.0 × 8 and 4.0 × 9 mm	LMCA-LAD	Yes	No
7	1	Drug-eluting	75 μm	3.5 × 23 mm	LMCA-LAD	Yes	No
8	1	Drug-eluting	65 μm	3.5 × 16 mm	LMCA-LAD	Yes	No
9	2	Drug-eluting+ Bare-metal	86 μm	4.0 × 15 and 4.0 × 12 mm	Isolated LMCA	Yes	No
10	1	Drug-eluting	81 μm	4.0 × 12 mm	Isolated LMCA	Yes	No
11	2	Drug-eluting+ Drug-eluting	75 μm	4.0 × 13 and 4.0 × 15 mm	Isolated LMCA	Yes	No
12	1	Drug-eluting	75 μm	4.0 × 16 mm	LMCA-LAD	Yes	No

IVUS: intravascular ultrasound; LAD: left anterior descending artery; LMCA: left main coronary artery.

**Figure 3:** – Coronary angiography and intravascular ultrasound before and after stent implantation in the left main coronary artery (patient 5). In the baseline assessment (A), marked luminal narrowing is observed both on coronary angiography (left) and intravascular ultrasound (right), showing a slit-like lumen without evidence of atherosclerotic plaque. After stent implantation (B), restoration of vessel lumen dimensions and symmetry is noted.

All patients experienced improvement in angina symptoms after coronary angioplasty, with complete symptom resolution in eight cases and significant relief in one patient. Beyond the first year, four patients underwent noninvasive reassessment of stent patency by CTA, which confirmed the maintenance of satisfactory results in all cases (Figure 4). This practice was applied on an individualized basis, according to the attending physician’s discretion.

**Figure 4:** – Coronary computed tomography angiography (patient 4) before and after angioplasty of left main coronary artery (LMCA) with stent implantation. In the upper row (A and B), pre-angioplasty images show significant luminal narrowing at LMCA origin due to extrinsic compression by the main pulmonary artery. In the lower row (C and D), post-angioplasty images demonstrate the stent covering the LMCA from the ostium, with preserved lumen and no signs of residual stenosis. Images were obtained using three-dimensional reconstruction (A and C) and curved reconstruction (B and D).

Dual antiplatelet therapy was maintained for 10±7 months, with no reported bleeding events. Over a mean follow-up of 33 months, escalation of specific PH therapy was required in 50% of patients due to disease progression. During follow-up, two patients died from PH decompensation (12 and 18 months after angioplasty), and two patients died at home from sudden cardiac death (7 and 47 months after angioplasty), all with PH progression and classified as intermediate or high risk at the time of PCI.

## Discussion

This study represents the first published case series in Brazil addressing the treatment of extrinsic LMCA compression in patients with PH. The prognosis of this condition remains poorly understood and is likely not directly comparable to that of atherosclerotic LMCA obstruction. Although it is a recognized cause of angina, its impact on mortality has not yet been clearly established. Considering that approximately 25% of deaths in patients with PH result from sudden cardiac death,^6,14^ some of these events may be related to extrinsic LMCA compression — a potentially treatable, likely underdiagnosed complication.

The interval between symptom onset and the diagnosis of PH is usually more than 2 years, during which time most patients already present with advanced disease.^19^ As a consequence, significant dilation of the pulmonary arteries is often observed. Due to their anatomical proximity, these arteries can displace the LMCA toward the coronary sinus, resulting in narrowing of its ostium. Studies have reported a prevalence of up to 40% of significant obstruction of LMCA due to extrinsic compression in patients with PH and angina.^8^ In a previous series from InCor, Mesquita et al. evaluated 36 patients with group 1 PH and congenital heart disease who underwent coronary angiography, identifying LMCA compression greater than 50% in seven cases. The main predictors of compression identified were a pulmonary artery diameter greater than 40 mm and a higher pulmonary artery-to-aorta ratio.^20^ Similar findings were reported by Galie et al., who described a sensitivity of 83% and specificity of 70% for the criterion of pulmonary artery diameter exceeding 40 mm.^8^ Additionally, Akbal et al. demonstrated that the severity of PH, assessed by higher mean pulmonary artery pressure and pulmonary vascular resistance, is also associated with an increased risk of LMCA compression.^15^ In the present series, only one patient had a pulmonary artery diameter below 40 mm, with the mean recorded diameter being 56 mm (ranging from 34 mm to 108 mm).

In 2001, Kajita et al. described the characteristic angiographic pattern of extrinsic LMCA compression, demonstrating a crushed appearance of the vessel between the pulmonary artery and the aortic root. In that study, one of the most widely used current diagnostic parameters was reported: the marked angulation of the LMCA origin, generally less than 39.5 degrees. Additionally, the left anterior oblique cranial projection was identified as the optimal view for angiographic assessment in these patients.^17^ This projection also provided the best diagnostic definition in our series, differing from the views typically used to evaluate atherosclerotic obstruction of LMCA.

Despite the existing reports, several gaps remain regarding the optimal management of these patients, given the still limited number of published cases. It is known that coronary artery bypass grafting in patients with PH carries a high mortality rate, mainly due to right ventricular dysfunction in the postoperative period. In this context, PCI has emerged as an effective therapeutic alternative for the treatment of extrinsic compression of LMCA.

Saia et al. published the largest available case series, describing 53 patients with PH and ≥50% obstruction of LMCA due to extrinsic compression who underwent coronary angioplasty.^16^ A satisfactory primary result was achieved in all procedures. Coronary angiography was routinely repeated after 9 months, with reintervention required in five patients: four due to neointimal hyperplasia and one due to stent recoil. During a mean follow-up of 4.5 years, the mortality rate was 37.3% (n=17), with no cases of acute myocardial infarction or stent thrombosis reported. In the present series, the observed mortality rate was 33.3%, which is comparable to that of the Italian series and consistent with the reported life expectancy in patients with PAH.^7^

The limitations of the present study include its observational design, without the inclusion of a control group, which limits the direct comparison of results with other therapeutic modalities or management strategies. Additionally, the small sample size, combined with the retrospective nature of the analysis and the relatively short follow-up period, does not allow for definitive conclusions regarding the durability of the results obtained or the incidence of long-term complications, such as stent restenosis. These limitations should be carefully considered when interpreting the findings. Future studies with larger patient populations and longer follow-up will be essential to clarify the prognostic impact of this therapeutic approach.

## Conclusion

Extrinsic compression of LMCA by pulmonary artery dilation should be considered as a differential diagnosis in patients with PH presenting with angina or left ventricular dysfunction. PCI has proven to be a feasible strategy in specialized PH centers, with favorable angiographic and clinical outcomes. However, additional studies are needed to assess the prognostic impact of this intervention and to clarify the potential benefits of systematic screening for coronary compression in asymptomatic patients.
